# A confusing case of canine vector-borne disease: clinical signs and progression in a dog co-infected with *Ehrlichia canis *and *Bartonella vinsonii *ssp. *berkhoffii*

**DOI:** 10.1186/1756-3305-2-S1-S3

**Published:** 2009-03-26

**Authors:** Edward B Breitschwerdt, Ricardo G Maggi

**Affiliations:** 1 Department of Clinical Sciences, College of Veterinary Medicine, North Carolina State University, 4700 Hillsborough Street, Raleigh, NC 27606, USA

## Abstract

*Bartonella *spp. are important pathogens in human and veterinary medicine, and bartonellosis is considered as an emerging zoonosis that is being reported with increasing frequency. Of 22 known species and subspecies of *Bartonella*, seven have been isolated from dogs, causing disease manifestations similar to those seen in human beings. The wide variety of clinical signs and the possible chronic progression of disease manifestations are illustrated in the case of an infected Labrador retriever. Here, the authors discuss the seemingly diverse spectrum of disease manifestations, the co-infections of *Bartonella *spp. with other vector-borne pathogens (mainly *Ehrlichia *spp. or *Babesia *spp.) and the difficulties in microbiological confirmation of an active *Bartonella *infection, all of which make the disease pathogenesis and clinical diagnosis more problematic.

## Findings

Historically, the most prevalent *Bartonella *species in dogs, *B. vinsonii *ssp. *berkhoffii*, was initially isolated from a dog with endocarditis and intermittent epistaxis in 1993 [1,2]. Subsequently, this pathogen has also been associated with cardiac arrhythmias, myocarditis, granulomatous rhinitis, anterior uveitis and chorioditis [1,3-6]. Other *Bartonella *species which have also been associated with pathology and clinical signs in dogs, including endocarditis, hepatic disease and sudden death are: *B. henselae *[7-11], *B. clarridgeiae *[9,12], *B. washoensis *[13], *B. elizabethae *[14] and *B. quintana *[15]. The first and the last, *B. henselae *and *B. quintana*, were also detected in the blood or lymph nodes of healthy dogs and dogs suffering from lymphoma [8]. More recent studies indicate that *B. henselae *may be the most frequent cause of bartonellosis in dogs.

The numerous disease manifestations in dogs, many of which are similar to those seen in humans, are listed in Table 1.

**Table 1:** *Bartonella *infection in dogs.

Species/subspecies	Associated manifestations of infection [23-31]
*Bartonella vinsonii *subsp. *berkhoffii*	Endocarditis*
	Cardiac arrhythmias*
	Myocarditis*
	Polyarthritis*
	Granulomatous rhinitis
	Anterior uveitis
	Chorioretinitis*
	Meningoencephalitis*
	Anaemia/Thrombocytopenia*
	
*Bartonella henselae*	Peliosis hepatitis*
	Granulomatous hepatitis*
	Generalised pyogranulomatous lymphadenitis*
	Panniculitis
	Endocarditis*
	Polyarthritis*
	Idiopathic effusions
	
Other *Bartonella *species	Endocarditis*
*Bartonella clarridgeiae*	Hepatic disease
*B. washoensis*	Weight loss
*B. elizabethae*	
*B. quintana*	

* Denotes disease manifestations reported in dogs and human beings.

*Bartonella *spp. are transmitted by bites and scratches of infected animals to other hosts. In addition, arthropod vectors may play the most important role in the transmission of these organisms. *B. henselae*, the agent of cat-scratch disease (CSD), has been isolated from cat fleas, and transmission by ticks has been proposed [16,17]. Although tick transmission of *B. vinsonii *ssp. *berkhoffii *has been suspected for over a decade based upon epidemiological evidence, this has not been proven [17]. Evidence suggests that dogs, coyotes and gray foxes may be the reservoir hosts for *B. vinsonii *ssp. *berkhoffii *[18,19]. Due to the mode of transmission a co-infection with other arthropod-borne pathogens is possible and may mask or alter the typical clinical signs of each of these pathogens. The following case of a chronically ill Labrador retriever is shortly outlined to illustrate the potential confusion and clinical challenges induced by tick-borne infections:

A 3-year-old, spayed, female Labrador retriever was referred to the North Carolina State University Veterinary Teaching Hospital for evaluation of a protracted illness that had begun approximately 9 months earlier. Initially, the dog was lethargic and intermittently inappetent and had a shifting-leg lameness suggestive of polyarthritis. On the initial examination prior to referral, the dog was mildly anaemic (haematocrit, 36%) and hyperproteinaemic (8.0 g/dl), but showed no obvious physical examination abnormalities. Two weeks later, the dog developed a grand mal seizure, accompanied by spontaneous urination. During the subsequent month, lethargy continued and weight loss was recorded (5.0 kg). On a second veterinary examination numerous intradermal haemorrhages on the neck and trunk were noticed as well as anaemia (haematocrit: 32%) with spherocytosis, mild thrombocytopenia (platelets: 180,000/μl), hyperglobulinaemia (serum globulin concentration: 4.7 g/dl), proteinuria, and hemoglobinuria (urine specific gravity: 1.034; 4+ protein; 4+ blood; 4 to 8 erythrocytes per high-power field) and physiological leukocyte counts and differential cell numbers. Seroreactivity to *Ehrlichia canis*, *Rickettsia rickettsii *and nuclear (antinuclear antibodies) antigens by indirect fluorescent-antibody assays (reciprocal titers, 50, 256 and 640, respectively) were detected. Ehrlichiosis and systemic lupus erythematosus were diagnosed by the referring veterinarian.

Treatment was initiated with tetracycline hydrochloride (750 mg 3 times daily for 14 days) and prednisone (40 mg twice daily (BID) for 3 days, then gradually tapered to 15 mg every other day).

During the next 3 months, episodes of listlessness and epistaxis occurred and on referral examination the dog was lethargic with slight tachycardia, a hyperdynamic arterial pulse accompanied by arterial pulse deficits and occasional premature beats. A diastolic and a systolic heart murmur were recorded. On electrocardiogram and spectral Doppler echocardiography extrasystoles and severe aortic as well as mild mitral valve insufficiency were diagnosed, already resulting an increase in interstitial and alveolar pulmonary infiltrates in the accessory lung lobe (thoracic radiographs). Laboratory parameters proved anaemia (haematocrit: 33%; mild anisocytosis and macrocytosis), thrombocytopenia (platelet count: 121,000/μl), leukocyte counts at 12,300/μl, with normal differential cell numbers, hypoalbuminaemia (serum albumin: 2.6 g/dl), azotaemia (blood urea nitrogen: 34 mg/dl) and hypokalaemia (serum potassium: 3.9 g/dl). Urinalysis again revealed proteinuria and haemoglobinuria (specific gravity: 1.011; 3+ protein; 2+ blood), haematuria (5 to 10 erythrocytes per high-power field), pyuria (5 to 10 leukocytes per high-power field) and bacteriuria. Aerobic and anaerobic blood cultures during a 24-h period as well as terminal subcultures and Gram stains performed after 7 days failed to grow or identify bacteria. Blood cultured simultaneously by the lysis centrifugation technique grew a fastidious, gram-negative organism. Seroreactivity (indirect fluorescent-antibody assay) to *E. canis *and *R. rickettsii *was again positive (reciprocal titers, 64 and 128, respectively) and a specific antibody response to *E. canis *was confirmed by Western immunoblot analysis.

Treatment for bacterial endocarditis accompanied by congestive heart failure consisted of enrofloxacin (306 mg BID), doxycycline (400 mg BID), clavulanate-potentiated amoxicillin (530 mg BID), furosemide (40 mg BID), digoxin (0.25 mg BID) and enalapril (20 mg BID). But because of a lack of substantial clinical improvement, intractable epistaxis and the poor long-term prognosis associated with vegetative valvular endocarditis, the owners elected euthanasia 17 days following discharge.

A lysis centrifugation technique finally isolated *B. vinsonii *ssp. *berkhoffii *genotype I in this dog by blood culture so that it was suggested that the dog was co-infected with *E. canis *and *B. vinsonii *ssp. *berkhoffii*. Reactivity to *Rickettsia *antigens is suggested to be cross-reactive with *Bartonella *antigens and anti-nuclear antibodies can occur with an increased frequency in dogs that are seroreactive to *B. vinsonii *ssp. *berkhoffii *and *E. canis *antigens [20].

The above-described case report highlights three problems related to infections with *Bartonella *spp.: (1) A large variety of disease manifestations makes a clinical diagnosis more difficult (diagnostic indications that support the testing for *Bartonella *infection are listed in Table 2); (2) co-infections of *Bartonella *spp. with other vector-borne pathogens particularly *Ehrlichia *spp. or *Babesia *spp. may influence the pathogenesis of those diseases and alternatively co-infection may also alter clinical signs of bartonellosis, creating substantial challenges for the clinician in regard to accurate diagnosis and directed medical management; (3) microbiological confirmation of an active *Bartonella *infection is difficult. Concerning the last point, and as is seen with other intracellular pathogens that induce chronic infection after vector-borne transmission, this aspect of diagnostic confirmation can be very challenging [7]. Ideally, diagnosis should be confirmed by culturing the organism from biopsy specimens, blood or fluid samples that are aseptically obtained. Due to the fastidious growth characteristics of *Bartonella *spp. it is important to avoid contamination of diagnostic specimens with skin flora or other rapidly growing bacteria. Unlike cats, which may easily have a bacterial load of 100,000 copies/μl, dogs are likely to have levels that are 100- to 1000-fold lower [7]. The presence of antibodies can only be used to infer prior exposure, but a substantial number of infected dogs do not have detectable antibodies. PCR amplification of *Bartonella *DNA following direct extraction from patient samples is also relatively insensitive. Two media have been developed to overcome the problem of slow-growing pathogens and unavailable liquid medium: "BAPGM" medium [21] and another medium developed by Riess *et al*. [22]. Nevertheless, culturing remains very time-consuming and when the need for a diagnosis is urgent, treatment should be started after obtaining appropriate diagnostic specimens. Serum antibodies can be detected in both healthy and clinically ill dogs and thus do not always correlate with illness. Therefore antibodies may only reflect prior exposure, but do not necessarily document that *Bartonella *is the cause of the current disease.

**Table 2:** Differential diagnostic indications that support consideration of testing for *Bartonella *infection.

Granulomatous inflammatory lesions
Unexplained reactive lymphadenopathy
Endocarditis
Myocarditis
Polyarthritis
Immune-mediated haemolytic anaemia
Immune-mediated thrombocytopenia
Eosinophilia
Splenomegaly
Epistaxis
Idiopathic cavitary effusions
Unexplained neurologic disease
Fever of unknown origin
Vasculitis
Chronic hepatitis

*Bartonella *spp. infections in dogs are increasingly reported. Dogs show a variety of clinical manifestations ranging from asymptomatic to severe disease, a fact that makes clinical diagnosis quite difficult. Studies have shown that infection with *B. vinsonii *ssp. *berkhoffii *can induce polyarthritis, seizures, vasculitis, epistaxis and endocarditis in dogs (all of which occurred in progression in the illustrated case report).

Other underlying co-infections which are also vector-borne, such as infections with *Ehrlichia canis *and *Babesia *spp., may further influence the clinical presentation and also influence the prognosis, particularly if all co-infected organisms are not detected or specifically treated. Finally, laboratory diagnosis of *Bartonella *infection in dogs can also be quite difficult and time-consuming (if performed by culturing). Many of these aspects could be illustrated in this case report of a confusing tick-borne disease and should be kept in mind by veterinary clinicians.

## Competing interests

Drs Breitschwerdt and Maggi are scientific officers for Galaxy Diagnostics, a newly formed company devoted to enhanced detection of *Bartonella *spp. infection in animals and human beings and located in Raleigh, NC, USA.

## Authors' contributions

Drs Breitschwerdt and Maggi have collaborated for the past five years on studies to enhance the diagnosis, treatment and prevention of *Bartonella *infection in animals and human beings. Each contributed equally to the content of this case report.
